# Social media-driven behavioral mechanisms for sustainable park governance: a analysis of visitation intentions

**DOI:** 10.3389/fpsyg.2025.1647976

**Published:** 2025-09-17

**Authors:** Rong Chen, Qi-tang Huang, Lv-lin Miao, Zheng Lin, Dong-dong Gao

**Affiliations:** ^1^Fuzhou Technology and Business University, Fuzhou, China; ^2^Fujian Agriculture and Forestry University, Fuzhou, China; ^3^Ningde Urban and Rural Planning Institute, Ningde, China

**Keywords:** social media usage, perceived attractiveness, visitation intention, perceived crowding risk, urban park

## Abstract

Social media has emerged as a critical driver of urban park visitation, reshaping public perceptions and behavioral patterns through information dissemination and emotional resonance. This study investigates how social media usage influences recreational visitors’ intentions to visit urban parks, integrating the Theory of Planned Behavior (TPB) and the Stimulus-Organism-Response (S-O-R) model. Data were collected via questionnaires distributed to 535 visitors across four mountain parks in Fuzhou, China. Structural equation modeling (SEM) and Bootstrap methods were employed to analyze the relationships between social media usage intensity, information quality, perceived attractiveness, perceived crowding risk, subjective norms, and visitation intention. Results demonstrated that social media usage intensity (*β* = 0.374, *p* < 0.001) and information quality (*β* = 0.175, *p* < 0.001) directly and significantly influenced visitation intention. Perceptual factors partially mediated these relationships: usage intensity indirectly enhanced behavioral intention by increasing perceived attractiveness and strengthening subjective norms, while information quality optimized decision-making by reducing perceived crowding risk. The findings highlight the dual pathways of rational cognition and emotional drive in shaping visitor behavior, underscoring the importance of balancing ecological protection and visitor experience in smart park management. This framework provides data-driven insights for sustainable park governance.

## 1 Introduction

The co-evolution of the digital technology revolution and the transformation of urban public spaces has deeply reshaped public perceptions and behavioral patterns toward urban parks through social media. Notably, social media-driven cultural and tourism consumption increasingly contributes to urban park visitation growth, with its proportional impact expanding significantly (Cheng et al., 2022). On social platforms, topics related to “viral check-in spots” have accumulated billions of views (Kui et al., 2021), reflecting a fundamental shift in public value cognition of landscape spaces—from traditional aesthetic appreciation to experience consumption dominated by traffic propagation metrics. The ongoing digital transformation engenders a dual-faceted challenge for landscape architecture as a discipline. Primarily, algorithmic recommendation systems generate ‘filter-enhanced landscapes’ that subvert the intentional spatial narratives crafted by design professionals, while concurrently, UGC actively reshapes place-based significations through psycho-emotional diffusion mechanisms and semiotic exchanges, thereby cultivating artificially constructed place affiliations that amplify recreational site appeal (Liu, 2021; Shang et al., 2023). Secondarily, the fundamental tension between ephemeral virality and the imperative for sustainable spatial governance reveals critical deficiencies within conventional design frameworks’ capacity to accommodate digital incursions. This systemic inadequacy manifests most prominently in the discipline’s constrained ability to dynamically respond to multimodal behavioral datasets—particularly those capturing temporal crowd density variations and affectively charged utilization patterns that require real-time spatial recalibration (Baksi and Sanyal, 2024). These contradictions reveal the core pain points of current urban park management: a dynamic balance mechanism has yet to be established between the spatial attractiveness empowered by algorithms and the sustainability of ecological carrying capacity. This study specifically targets this critical gap, decoding the ‘cognitive-behavior’ black box (Alexander, 2025) triggered by social media, to provide theoretical anchors and practical paths for precise park governance in the digital age.

Current research continues to face cognitive gaps in interdisciplinary integration and systematic framework development, a phenomenon warranting heightened scholarly attention. Preliminary survey data reveal that 73% of urban residents acquire park information through social platforms (WeChat Moments, Xiaohongshu, Douyin), compared to only 18% relying on official guidance systems, with exposure intensity to UGC (≥30 min daily) demonstrating a significant positive correlation with visitation intentions (*r* = 0.41, *p* < 0.01). Notably, while UGC exhibits higher contextual empathy indices than official sources (Xiang and Gretzel, 2010; Liu et al., 2020), its information overload induces decision-making risks, frequently causing visitor dissatisfaction due to geolocation tagging inaccuracies (Law et al., 2010; Park and Nicolau, 2015). This paradox stems from theoretical underdevelopment: existing studies neither quantify the perceptual diminution of landscape authenticity caused by short-video montage narratives (Wu and Lai, 2024; Yin et al., 2024), nor systematically verify differentiated mechanisms of information types within the “perception-behavior” transmission chain.

Current academic research in this field faces two critical developmental bottlenecks requiring scholarly attention. First, at the theoretical level, the adaptation of the Theory of Planned Behavior (TPB) to tourism contexts inadequately addresses the low-involvement decision-making characteristics of daily leisure behaviors, while the influence mechanisms of social media information on attitude formation remain insufficiently integrated into the theoretical framework (Tan, 2020). Second, at the analytical framework level, constrained by the linear logic of the Stimulus-Organism-Response (S-O-R) model, studies struggle to decipher the nonlinear mediation pathways between informational stimuli (X), perceptual processing (O), and behavioral responses (R), particularly in hybrid digital-physical decision-making environments. This limitation hinders the identification of threshold effects in UGC exposure intensity and feedback loops arising from geolocation inaccuracies, thus obscuring complex interactions between algorithmic recommendations and spatial behavior patterns.

This investigation responds to these disciplinary challenges through methodological innovation employing a multi-mediating chain structural equation modeling (SEM) framework. At the applied dimension, the research introduces a novel “credibility-attractiveness” adaptive equilibrium framework, providing theoretically grounded solutions for optimizing data governance protocols in intelligent green space systems. Theoretically, it systematically investigates the operational mechanisms through which digitally mediated landscape representations condition visitation motivations via dual-mediating pathways: psycho-emotional engagement processes and informational trust calibration mechanisms. This dual analytical approach advances both pragmatic management strategies and conceptual understanding of digital-physical spatial interactions in contemporary landscape systems.

## 2 Theoretical framework and model construction

This study integrates the Theory of Planned Behavior (TPB) with the Stimulus-Organism-Response (S-O-R) model to construct a theoretical framework. The Theory of Planned Behavior (Liu et al., 2025) posits that behavioral intention is determined by behavioral attitude (individual evaluation of the behavior), subjective norm (perceived social expectations), and perceived behavioral control (assessment of the ease or difficulty of performing the behavior). Although widely applied in tourism decision-making research (Woosnam et al., 2024), this theory has limitations in explaining the influence path of digital media (such as algorithm recommendations). The Stimulus-Organism-Response model (Qiao et al., 2024) suggests that environmental stimuli (such as characteristics of social media content) trigger individual cognitive and emotional states (such as risk perception), ultimately leading to behavioral responses (such as visitation behavior), providing an effective path for analyzing the impact of digital content on behavior (Tam et al., 2024; Li et al., 2025). This study constructs an integrated model of ‘Social Media Information-Cognition-Behavior’ by combining the normative cognitive system of TPB with the environmental psychological mechanism of S-O-R, retaining TPB’s explanatory power for intention formation while enhancing the dynamic analytical capability of digital media characteristics. The theoretical contributions are articulated as follows:

Extension of TPB Model: The research incorporates multi-dimensional social media informational taxonomies as exogenous determinants, establishing parallel mediation routes via epistemic trust validation mechanisms and affective resonance processes. This dual-channel mediation architecture reconceptualizes the formative dynamics of behavioral intentionality constructs – specifically recalibrating attitude formation matrices (ATT) and socio-cultural normative evaluations (SN)—through digital information metabolization pathways, thereby advancing theoretical precision in technology-mediated behavioral prediction models.

Elaboration of S-O-R Model: The stimulus component (S) is rigorously conceptualized as polymorphic information carrier attributes, encompassing modality-specific presentation formats and provenance authentication indicators. The organism dimension (O) delineates a psychological architecture comprising perceptual distinctiveness appraisals (PU) and anticipatory risk assessment schemata (PR). Response manifestations (R) are dichotomized into behavioral response modalities: destination engagement propensity (VI) and participatory dissemination propensity (SW), thereby operationalizing granular outcome differentiation in digital-physical space interaction phenomena. This tripartite specification establishes measurable linkages between informational stimuli characteristics, cognitive-affective processing systems, and differentiated behavioral commitment patterns in hybridized spatial contexts.

### 2.1 Theoretical framework

Social cognitive theory posits that social media, as an information medium, directly shapes users’ destination perceptions (e.g., landscape attractiveness, facility convenience) through high-frequency, multimodal content exposure (e.g., scenic images, short videos) (Filieri et al., 2015). For instance, exposure to aesthetically curated destination visuals on social media can amplify users’ cognitive appraisal of scenic value. From a technology acceptance model (TAM) perspective, social media usage intensity may enhance users’ perceptions of information credibility and platform interactivity, thereby reinforcing functional trust in destinations. Platform usability, for example, can mitigate risk perception and foster dependency (Escobar-Rodríguez and Carvajal-Trujillo, 2014). Cognitive psychology frameworks (e.g., information processing theory) further reveal that immersive social media features (e.g., VR previews, user-generated content) activate perceptual associations through direct sensory stimulation, bypassing mediating variables (Tussyadiah et al., 2018). Collectively, these mechanisms support Hypothesis 1 (H1):

*H1*: Social media usage exerts a direct, significant positive effect on perceptual factors.

The formation of tourists’ behavioral intention is driven by multiple perceptual factors, primarily manifested through a dual-path framework of cognitive rationality and emotional drive. Firstly, the cognitive rationality path, grounded in the Theory of Planned Behavior (TPB), indicates that tourists’ cognitive perceptions of destination attributes (such as perceived attractiveness, hedonic value, safety risks, crowding risks) constitute the core dimensions of behavioral attitude, directly influencing behavioral intention (Han and Kim, 2010). Specifically, tourists’ positive cognitive evaluations of destination value (e.g., hedonic, educational value) can foster positive attitudes and travel motivation. Concurrently, the risk-benefit trade-off model reveals that negative perceptions of risk (e.g., safety risks, crowding risks) undermine behavioral confidence, while clear destination information can indirectly facilitate decision-making by reducing uncertainty and enhancing perceived behavioral control (Fuchs and Reichel, 2011; Munoz-Mazon et al., 2021). Secondly, the emotional drive path, based on emotional attachment theory, shows that affective perceptions triggered by destination attributes (such as emotional resonance and aesthetic experiences evoked by landscape uniqueness) can directly stimulate tourists’ attachment to the place (e.g., place identity, sense of belonging), and this emotional bond itself translates into strong behavioral intentions (e.g., revisit intention, recommendation intention) (Yuksel et al., 2010).

Crucially, the cognitive rationality path and the emotional drive path are not isolated but interconnected, exerting a synergistic influence on behavioral intention. Rational cognitive appraisal of destination value and risk often serves as a prerequisite for forming positive emotional experiences (e.g., pleasure, excitement) and deep place attachment. For instance, recognizing the unique value of a landscape (cognition) makes awe and emotional resonance (emotion) more likely. Once a strong place attachment is formed, this emotional bond can not only directly drive behavioral intention but also reinforce the intention shaped by cognitive appraisal, even moderating sensitivity to negative cognitive factors (e.g., mild crowding, cost). For example, tourists with deep emotional ties to a place may retain strong visitation intention despite perceiving certain risks or costs. Ultimately, tourists’ behavioral intention results from the combined influence of cognitive appraisal (attitude, subjective norms, perceived control) and emotional attachment, where cognitive factors provide the rational basis for decision-making, and emotional factors inject motivational energy and loyalty.

Based on the above cognitive rationality path, emotional drive path, and their synergistic interaction mechanism, this study proposes Hypothesis 2 (H2):

*H2*: Perceptual factors exert a direct, significant positive effect on behavioral intentions.

Dual-process theories posit that social media usage may directly trigger behavioral intentions through automated pathways (e.g., user habits, platform dependency) without cognitive mediation (Chaiken, 1980). For instance, prolonged exposure to destination content can create behavioral inertia, leading to impulse decisions (“see-and-go” behavior). Social identity theory further suggests that identity markers on social media (e.g., “travel influencer” tags) may directly constrain user behavior through social commitment mechanisms, driving visitation to maintain one’s digital persona (Brown and Hewstone, 2005; Hogg and Terry, 2000). The stimulus–response (S-R) model reveals that algorithmic recommendation systems create filter bubble effects, directly increasing exposure frequency to specific destinations and inducing behavioral responses (Chaney et al., 2018). These mechanisms collectively support Hypothesis 3 (H3):

*H3*: Social media usage exerts a direct, significant positive effect on behavioral intentions.

### 2.2 Theoretical model construction

Building upon the environmental psychology S-O-R framework, external stimuli (S) can elicit affective responses (O) in individuals, subsequently influencing behavioral responses (R). Cognitive appraisal typically shapes affective valence, which collectively with cognitive evaluation determines emotional states that mediate responses to stimuli. In this process, the organism’s affective response may serve as a mediating mechanism.

**Figure 1:**
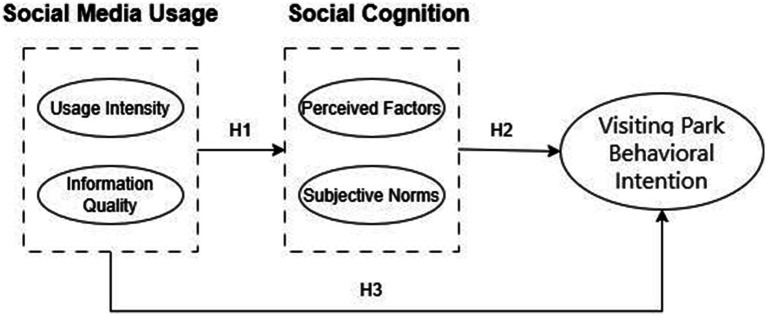
Theoretical structural relationship model.

Accordingly, we operationalize information carrier characteristics as stimulus factors that influence individuals through perceptual attributes, shaping subjective norms and ultimately generating visitation intentions. Integrating these theoretical propositions, we develop a structural relationship model of “social media usage → social cognition → visiting park behavioral intention” as illustrated in Figure 1.

## 3 Methodology

### 3.1 Research plot

**Figure 2:**
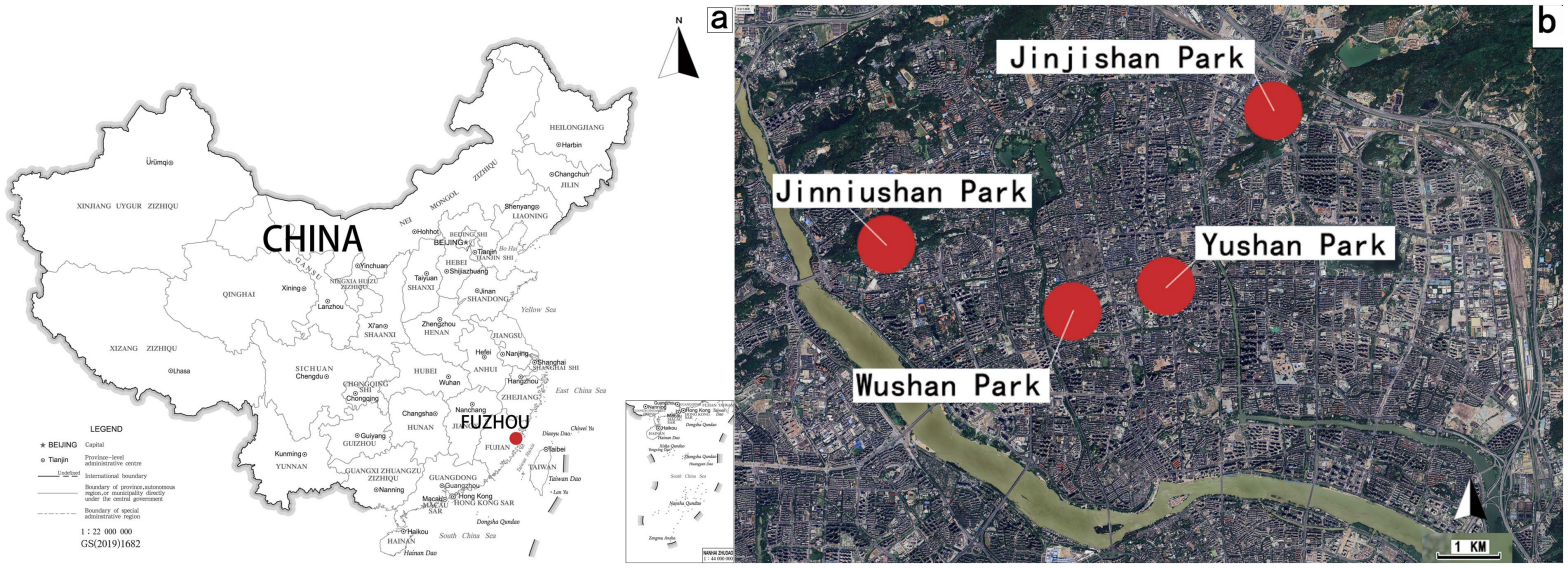
**(a)** Location of Fuzhou city in China map; **(b)** four Parks in Fuzhou city.

The research sites were selected from urban mountain parks in Fuzhou City, Fujian Province, eastern China. As a representative mountainous city, Fuzhou’s geographical attributes have shaped its distinctive urban form characterized by “mountains within the city and the city nestled among mountains,” which fundamentally influences its developmental patterns (Chen et al., 2023). Mountain parks, distinguished by their complex topography and abundant natural resources, serve as critical components of urban green infrastructure by providing three-dimensional and diverse recreational spaces while maintaining robust ecological foundations. However, these parks also face amplified challenges in planning, environmental maintenance, and management due to terrain constraints. Four representative parks (Wushan Park, Jinjishan Park, Yushan Park, and Jinniushan Park) were systematically selected based on geographical distribution, as shown in Figure 2, visitor flow, and establishment history to comprehensively reflect Fuzhou’s urban mountain park development.

### 3.2 Analytical approach

This investigation employs a multi-phase analytical strategy to empirically examine the interconnected dynamics between digital engagement patterns and spatial behavioral intentions within urban green space systems. The research framework necessitates simultaneous examination of cross-dimensional independent variables and heterogeneous outcome measures, creating analytical complexities that conventional multivariate regression techniques inadequately address due to their limitations in modeling multi-level variable interdependencies. Structural Equation Modeling (SEM) was strategically selected as the principal analytical methodology through rigorous methodological evaluation, leveraging its capacity for latent variable modeling and covariance structure analysis through three systematically implemented phases.

Initial exploratory procedures involved preliminary survey instrumentation to collect foundational response data, followed by exploratory factor analysis (EFA) techniques to dimensionalize latent constructs, establish measurement metrics, and refine psychometric survey tools. The operational phase executed formalized data collection through validated survey dissemination, with subsequent data processing utilizing the SPSS 27.0 platform for foundational diagnostics encompassing sample demographic profiling through descriptive statistics and reliability verification via internal consistency metrics. The confirmatory phase implemented advanced analytical protocols through AMOS 24.0 software, conducting validation testing that included confirmatory factor analysis (CFA) for construct validity assessment, followed by comprehensive evaluation of structural pathway relationships through model fit indices and parameter estimation techniques. This phased analytical architecture ensures methodological rigor in addressing complex mediational relationships inherent in digital-physical behavioral interfaces while maintaining statistical robustness across heterogeneous variable interactions.

#### 3.2.1 Exploratory factor analysis (EFA)

This method is often applied in the initial stage of research. Given the limited existing studies in academia regarding the dimensional division of landscape perception, it is necessary to identify the essential correlations among multiple variables by constructing a correlation coefficient matrix between multiple latent variables. This process synthesizes variables with intricate relationships into a few core factors, while also validating the rationality of theoretical variable grouping, thereby providing a scientific foundation for subsequent model construction research. Therefore, EFA was selected to explore the dimensions of landscape perception and construct a landscape perception scale. The specific scoring function for common factors is shown in Equation 1:


(1) $$F_{i}=\beta_{i1}X_{1}+\beta_{i2}X_{2}+\ldots +\beta_{\text{in}}X_{n},i=1,2,\ldots ,m$$

In Equation 1, *F_i_* represents a common factor; *β_in_* denotes the weight of each questionnaire evaluation variable, indicating the importance of each variable in the equation. The number of common factors is denoted by *m*, and the number of original variables in the measurement items is denoted by *n*.

#### 3.2.2 Structural equation modeling (SEM)

This method can not only conduct scientific reliability and validity tests on scales but also effectively handle complex relationships among multiple variables. SEM integrates measurement models and structural models, representing a robust integration of factor analysis and path analysis. It can measure different effects between multiple variables and estimate the overall model fit to verify the scientific rationality of the model. Among these, the measurement model analyzes the relationship between latent variables and observed variables, with its equations shown in Equations 2, 3.


(2) $$X=\Lambda_{x}\xi +\delta$$

(3) $$Y=\Lambda_{y}\eta +\varepsilon$$

In Equations 2, 3, *X* and *Y* represent exogenous observed variables and endogenous observed variables, respectively; *Λ_x_* and *Λ_y_* denote their respective factor loading matrices; *ξ* represents exogenous latent variables; *η* represents endogenous latent variables; *δ* and *ε* represent measurement error vectors.

The structural equation model is shown in Equation 4:


(4) $$\eta =B_{\eta }+\Gamma \xi +\varsigma$$

In Equation 4, *B* and *Γ* represent the coefficient matrices of endogenous latent variables and exogenous latent variables, respectively; *ξ* denotes exogenous latent variables; *ζ* represents the random error term.

### 3.3 Measures

The questionnaire in this paper consists of four parts: social demographic variables, social media usage scale, social cognition scale, and park visit behavioral intention scale. Except for the demographic variables, the remaining three scales were developed and designed in conjunction with the research site, specifically divided into three steps: (1) Synthesizing relevant literature studies to organize and summarize measurement items; (2) Formulating some items based on corresponding theoretical concepts and integrating online text evaluations to supplement the deficiencies of existing studies; (3) Consulting experts in landscape architecture and related fields, distributing expert questionnaires, eliminating some unreasonable items, and further improving the questionnaire. Through the above research steps, the measurement scale was initially constructed. The reliability and validity of the scale were tested using data obtained from offline questionnaire collection. A total of 150 questionnaires were distributed, and 150 valid questionnaires were recovered.

The test results, as shown in Table 1, indicate that the KMO value is 0.887, exceeding the recommended threshold of 0.7, and the significance level of Bartlett’s Test of Sphericity is 0.00 (*p* < 1%), suggesting that factor analysis is appropriate. Results of principal component extraction from the 26-item scale showed that six factors with initial eigenvalues greater than 1 were extracted, accounting for a cumulative explained variance of 67.442%. Specifically, Factor 1 had an eigenvalue of 6.427 (explaining 24.719% of variance), Factor 2 had an eigenvalue of 3.634 (13.976%), Factor 3 had an eigenvalue of 2.557 (9.834%), Factor 4 had an eigenvalue of 2.17 (8.344%), Factor 5 had an eigenvalue of 1.609 (6.189%), and Factor 6 had an eigenvalue of 1.139 (4.38%).

**Table 1:** KMO and Bartlett test.

KMO sampling adequacy statistic		0.887
Bartlett’s test of sphericity	chi-square approximation	6495.773
	df	325
	Sig.	0.000

Factor assignments were determined using the rotated component matrix: Items YJ3, YJ2, YJ5, YJ1, and YJ4 were assigned to Factor 1 (all factor loadings exceeding 0.7) and labeled “Perceived Crowding Risk” based on item content; Items XYL2, XYL4, XYL1, XYL5, and XYL3 were assigned to Factor 2 (all factor loadings > 0.7) and named “Perceived Park Attractiveness”; Items YX2, YX1, YX3, and YX4 were assigned to Factor 3 (all factor loadings > 0.7) and labeled “Visiting Park Behavioral Intention”; Items ZL2, ZL3, ZL1, and ZL4 were assigned to Factor 4 (all factor loadings > 0.7) and named “Information Quality”; Items GF3, GF2, GF1, and GF4 were assigned to Factor 5 (all factor loadings > 0.7) and labeled “Subjective Norms”; Items SY1, SY2, SY4, and SY3 were assigned to Factor 6 (all factor loadings > 0.7) and named “Usage Intensity.” The study adopted a 5-point Likert scale ranging from “Strongly Disagree” to “Strongly Agree.”

### 3.4 Data collection

Field surveys employing an offline convenience sampling method were conducted from January to February 2025, targeting recreational visitors within these parks. Prior to questionnaire administration, researchers provided concise explanations of survey items to ensure participants’ comprehension. The initial sample size was determined based on the 10:1 rule of thumb in SEM, which recommends a minimum sample size of 10 times the number of observed variables (Hair, 2009). Given that the measurement model included 33 observed variables, the targeted sample size of 330 was exceeded, further enhancing the statistical power and reliability of the model estimation.

A total of 550 questionnaires were distributed, with 535 valid responses retained after quality screening, yielding a high valid response rate of 97.27%.

### 3.5 Ethical considerations

This study was conducted in compliance with established ethical guidelines for human subjects research, following the standards set forth by the American Psychological Association (Roche, 2024). Before data collection commenced, the research protocol underwent formal review and received approval from the Institutional Review Board (IRB) to ensure alignment with federal human research protections. Participants were provided with detailed information regarding voluntary involvement and were assured that all collected data would remain strictly confidential through anonymized processing. The questionnaire design underwent rigorous review to exclude any items potentially causing psychological harm or distress, aligning with APA’s emphasis on minimizing risks in research involving human subjects. Participants were appropriately acknowledged for their time and contributions through standardized debriefing procedures (see Tables 2, 3).

**Table 2:** Total variance explained.

Component	Initial eigenvalues			Extracted sum of squared loadings			Rotated sum of squared loadings		
	Total	Percentage variance %	Cumulative %	Total	Percentage variance %	Cumulative %	Total	Percentage variance %	Cumulative %
1	6.427	24.719	24.719	6.427	24.719	24.719	3.378	12.991	12.991
2	3.634	13.976	38.695	3.634	13.976	38.695	3.321	12.772	25.763
3	2.557	9.834	48.529	2.557	9.834	48.529	2.807	10.795	36.557
4	2.17	8.344	56.873	2.17	8.344	56.873	2.739	10.536	47.093
5	1.609	6.189	63.062	1.609	6.189	63.062	2.68	10.308	57.402
6	1.139	4.38	67.442	1.139	4.38	67.442	2.61	10.04	67.442
7	0.668	2.569	70.011						
8	0.581	2.235	72.245						
9	0.551	2.12	74.365						
10	0.525	2.019	76.384						
11	0.519	1.996	78.38						
12	0.488	1.878	80.258						
13	0.473	1.821	82.079						
14	0.45	1.73	83.809						
15	0.447	1.719	85.528						
16	0.425	1.636	87.164						
17	0.41	1.577	88.741						
18	0.396	1.523	90.263						
19	0.375	1.441	91.704						
20	0.357	1.374	93.078						
21	0.348	1.34	94.418						
22	0.33	1.268	95.686						
23	0.303	1.165	96.851						
24	0.289	1.113	97.964						
25	0.274	1.055	99.019						
26	0.255	0.981	100						

**Table 3:** The rotated component matrix.

Title	Component					
	1	2	3	4	5	6
YJ3	0.797					
YJ2	0.793					
YJ5	0.791					
YJ1	0.783					
YJ4	0.783					
XYL2		0.805				
XYL4		0.804				
XYL1		0.79				
XYL5		0.789				
XYL3		0.769				
YX2			0.788			
YX1			0.787			
YX3			0.778			
YX4			0.762			
ZL2				0.805		
ZL3				0.8		
ZL1				0.79		
ZL4				0.771		
GF3					0.84	
GF2					0.79	
GF1					0.78	
GF4					0.772	
SY1						0.775
SY2						0.775
SY4						0.738
SY3						0.723

## 4 Results and analysis

### 4.1 Common method bias

The sample characteristics in Table 4 reveal the following features: Gender distribution was balanced, with females accounting for 51.8% (277 respondents), slightly higher than males (48.2%). The age structure showed high concentration, with the 26–35 age group comprising over half of respondents (53.8%, 288 respondents), forming the core sample group. Income levels displayed a spindle-shaped distribution, where respondents with monthly disposable income of 6,001–10,000 yuan accounted for 32.7% (175 respondents) and those earning 3,001–6,000 yuan represented 30.7% (164 respondents), together constituting over 60% of the sample. Internet usage patterns were prominent: 97% of respondents used WeChat Moments for information access, while nearly half (48.4%) also used Douyin (TikTok). Engagement with park-related content was frequent, with 28.2% (151 respondents) viewing such content three to five times weekly and 10.7% (57 respondents) browsing multiple times daily. This sample profile reflects the characteristics of a young, middle-income, highly digitally active urban population group.

**Table 4:** Sample characteristic distribution description.

Variable	Options	Frequency	Percentage
Gender	Male	258	48.2%
	Female	277	51.8%
Age	Under 18	1	0.2%
	18–25 years old	129	24.1%
	26–35 years old	288	53.8%
	36–45 years old	92	17.2%
	46 years old and above	25	4.7%
Monthly disposable income (including students’ living expenses)	Below 3,000 yuan	85	15.9%
	3,001–6,000 yuan	164	30.7%
	6,001–10,000 yuan	175	32.7%
	Above 10,001 yuan	111	20.7%
Social media apps	WeChat moments	421	97.0%
	Douyin	259	48.4%
	Weibo	10	1.9%
	Dianping	21	3.9%
	Xiaohongshu	206	38.5%
	Others	110	25.3%
Frequency of browsing park-related content	Multiple times a day	57	10.7
	Once a day	66	12.3%
	1–2 times a week	138	25.8%
	3–5 times a week	151	28.2%
	Occasionally	123	23.0%

In Table 5, the analysis of valid survey data reveals key findings across six variable dimensions. Overall, visitors’ evaluations of park attributes ranged between moderate to slightly positive levels. Regarding social media usage, visitors showed high dependency on information acquisition (Item A4: *M* = 3.72 ± 1.008) but lower recognition of content breadth and utility—“multi-aspect information coverage” (A6) scored only 3.21 ± 0.882 while “information helpful for trip planning” (A8) reached 3.40 ± 1.032, indicating fragmented social media content. Social cognition displayed polarization: “activity entertainment value” (B2) emerged as the highest-scoring item (3.95 ± 0.841), whereas “landscape impressiveness” (B1) scored lower (3.41 ± 0.981) and “cultural background interest” (B5) reached 3.54 ± 0.968, suggesting room for improvement in landscape design and cultural storytelling. Notably, crowding risk perception was prominent (“crowding reduces comfort” B8: 3.87 ± 0.966), driving visitors to actively choose off-peak visits (B9: 3.68 ± 1.013). The subjective norms dimension revealed that social pressure (B14: 3.58 ± 0.889) exerted stronger influence than personal recommendations (B11: 3.34 ± 0.950), highlighting group identity’s role in visitation decisions. For behavioral intentions, time commitment willingness (“make time to visit” C3: 3.85 ± 0.911) exceeded financial commitment (“pay transportation costs” C4: 3.45 ± 0.993), though overall behavioral conversion remained limited (no item exceeded 4.00). These findings suggest the park should prioritize: (1) enhancing multidimensional integration of social media content (particularly landscape/cultural interpretation), (2) optimizing peak-period crowd management, and (3) upgrading visual design and cultural experiences to break through the current evaluation plateau.

**Table 5:** Statistical analysis of sample data.

Latent variable	Item	Symbol	Mean	Std. dev.	Min	Max
Social media usage		A				
Usage intensity	I browse social media content related to the park at least once a day.	A1	3.55	1.035	1	5
	I actively search for tags or keywords related to the park.	A2	3.44	1.054	1	5
	I have collected/shared posts related to the park.	A3	3.35	1.051	1	5
	I rely on social media to get the latest information about the park.	A4	3.72	1.008	1	5
Information quality	I think the park information on social media is accurately described.	A5	3.53	1.061	1	5
	The park information on social media covers multiple aspects (such as landscape, facilities, activities).	A6	3.21	0.882	1	5
	The park information on social media is updated in a timely manner.	A7	3.56	0.998	1	5
	The park information on social media helps me plan my visit.	A8	3.40	1.032	1	5
Social cognition		B				
Perceived park attractiveness	The landscape design of this park amazes me.	B1	3.41	0.981	1	5
	The activities in the park are interesting and participatory.	B2	3.95	0.841	1	5
	The environmental atmosphere of the park makes me feel relaxed and comfortable.	B3	3.43	1.067	1	5
	The park’s landscape design is visually striking, suitable for taking photos and check-ins.	B4	3.66	0.907	1	5
	The cultural or historical background of the park interests me.	B5	3.54	0.988	1	5
Perceived crowding risk	I worry that too many tourists in this park will affect my visiting experience.	B6	3.76	0.93	1	5
	Too many tourists will make it difficult for me to find a suitable photo spot.	B7	3.25	1.018	1	5
	I worry that crowding will reduce my visiting comfort in the park.	B8	3.87	0.966	1	5
	I prefer to visit the park during periods with less foot traffic.	B9	3.68	1.013	1	5
	I think the park’s tourist management measures can effectively relieve crowding.	B10	3.67	0.94	1	5
Subjective norms	My relatives and friends strongly recommend me to visit this park.	B11	3.34	0.95	1	5
	Recommendations on social media make me feel the park is worth visiting.	B12	3.49	1.024	1	5
	I choose this park based on others’ evaluations and recommendations.	B13	3.46	1.023	1	5
	Not going to this park may make me seem “left behind” or “not gregarious.”	B14	3.58	0.889	1	5
Visiting park behavioral intention	I am willing to choose this park as the first choice for weekend outings.	C1	3.77	0.846	1	5
	I will recommend others to visit this park.	C2	3.49	1.015	1	5
	Even if busy, I will find time to visit this park.	C3	3.85	0.911	1	5
	I am willing to bear the transportation cost to participate in the park activities.	C4	3.45	0.993	1	5

### 4.2 Reliability and validity tests and confirmatory factor analysis

After collecting research data through field questionnaires, we used AMOS 24.0 to conduct reliability and validity tests, confirmatory factor analysis, model goodness-of-fit analysis, hypothesis testing, and mediation effect analysis on the survey data from 535 valid questionnaires.

The statistical analysis was conducted using SPSS 29.0 to evaluate the reliability of the measurement model. As presented in Table 6, the internal consistency of the six constructs—measuring social media engagement, social cognition, and park visitation motivation—demonstrated strong reliability, with Cronbach’s *α* coefficients ranging between 0.806 and 0.898. The composite reliability score for the entire instrument reached 0.867, surpassing the recommended threshold of 0.7. As shown in Table 7, Furthermore, the dataset exhibited appropriate characteristics for factor analysis, as evidenced by a KMO measure of sampling adequacy (0.887) and statistically significant Bartlett’s test results (*p* < 0.01).

**Table 6:** Results of reliability, validity, and confirmatory factor analysis.

Latent variable	Number	Item	Standardized factor loadings	Average variance extracting (AVE)	Composite reliability (CR)	Cronbach’s alpha
Social media usage						
Usage intensity	1	I browse social media content related to the park at least once a day.	0.739	0.5104	0.8065	0.806
	2	I actively search for tags or keywords related to the park.	0.701			
	3	I have collected/shared posts related to the park.	0.71			
	4	I rely on social media to get the latest information about the park.	0.707			
Information quality	1	I think the park information on social media is accurately described.	0.772	0.5576	0.8344	0.832
	2	The park information on social media covers multiple aspects (such as landscape, facilities, activities).	0.753			
	3	The park information on social media is updated in a timely manner.	0.739			
	4	The park information on social media helps me plan my visit.	0.722			
Social cognition						
Perceived park attractiveness	1	The landscape design of this park amazes me.	0.741	0.5598	0.8641	0.862
	2	The activities in the park are interesting and participatory.	0.746			
	3	The environmental atmosphere of the park makes me feel relaxed and comfortable.	0.768			
	4	The park’s landscape design is visually striking, suitable for taking photos and check-ins.	0.767			
	5	The cultural or historical background of the park interests me.	0.718			
Perceived crowding risk	1	I worry that too many tourists in this park will affect my visiting experience.	0.75	0.5614	0.8649	0.864
	2	Too many tourists will make it difficult for me to find a suitable photo spot.	0.763			
	3	I worry that crowding will reduce my visiting comfort in the park.	0.74			
	4	I prefer to visit the park during periods with less foot traffic.	0.732			
	5	I think the park’s tourist management measures can effectively relieve crowding.	0.761			
Subjective norms	1	My relatives and friends strongly recommend me to visit this park.	0.734	0.5422	0.8255	0.824
	2	Recommendations on social media make me feel the park is worth visiting.	0.738			
	3	I choose this park based on others’ evaluations and recommendations.	0.777			
	4	Not going to this park may make me seem “left behind” or “not gregarious.”	0.694			
Visiting park behavioral intention	1	I am willing to choose this park as the first choice for weekend outings.	0.819	0.6933	0.9004	0.898
	2	I will recommend others to visit this park.	0.818			
	3	Even if busy, I will find time to visit this park.	0.853			
	4	I am willing to bear the transportation cost to participate in the park activities.	0.84			
Overall	1					0.867

**Table 7:** KMO test and significance results table.

Number	Latent variable	KMO	Significance
1	Usage intensity	0.801	<0.001
2	Information quality	0.808	<0.001
3	Perceived park attractiveness	0.875	<0.001
4	Perceived crowding risk	0.873	<0.001
5	Subjective norms	0.810	<0.001
6	Visiting park behavioral intention	0.847	<0.001
7	Total	0.887	0.000

The measurement model was validated using AMOS 24.0 through confirmatory factor analysis in Table 6. All observed indicators demonstrated strong relationships with their respective constructs, with standardized factor loadings ranging from 0.694 to 0.892, well above the recommended 0.5 threshold. The model exhibited excellent psychometric properties, as evidenced by composite reliability (CR) scores between 0.807 and 0.913, satisfying the 0.7 benchmark for internal consistency. Furthermore, convergent validity was confirmed with average variance extracted (AVE) values exceeding 0.510 across all constructs, meeting the minimum 0.5 criterion for adequate discriminant validity.

In Table 8, the chi-square/degrees of freedom (*χ*^2^/df) ratio is 1.313, which is less than 3, indicating an ideal fit. The RMSEA value is 0.024, below the 0.05 threshold, demonstrating an ideal fit. The GFI value of 0.950 exceeds 0.9, showing good model fit. Similarly, the AGFI value of 0.938 is above 0.9, confirming good fit. The CFI value of 0.986 is greater than 0.9, indicating excellent fit. The IFI value of 0.986 also surpasses 0.9, suggesting strong fit. The TLI value of 0.984 is above 0.9, verifying good model fit. Overall, the structural model incorporating social media usage, social cognition, and park visitation intention demonstrates excellent goodness-of-fit across all evaluated indices, with all parameters significantly exceeding established benchmarks and confirming the model’s robustness.

**Table 8:** Overall fitting coefficients table.

*χ*^2^/df	RMSEA	NFI	RFI	IFI	TLI	CFI	GFI	AGFI
1.313	0.024	0.944	0.935	0.986	0.984	0.986	0.950	0.938

Table 9 shows that significant correlations exist among usage intensity, information quality, attractiveness, crowding risk, subjective norms, and behavioral intention (*p* < 0.001). Furthermore, the absolute values of all correlation coefficients are below 0.5 and smaller than the square roots of their corresponding AVE values. This indicates that while the latent variables demonstrate certain intercorrelations, they also maintain adequate discriminant validity. The results confirm that the measurement scale exhibits ideal discriminant validity, as the constructs are sufficiently distinct from one another while remaining theoretically related.

**Table 9:** Discriminant validity.

Item	Usage intensity	Information quality	Perceived park attractiveness	Perceived crowding risk	Subjective norms	Visiting park behavioral intention
Usage intensity	0.510					
Information quality	0.031***	0.558				
Perceived park attractiveness	0.03***	0.027	0.560			
Perceived crowding risk	0.027	0.03***	0.025	0.561		
Subjective norms	0.025***	0.025***	0.023	0.023	0.542	
Visiting park behavioral intention	0.038***	0.036***	0.032***	0.033***	0.029***	0.693
Square root of AVE	0.714	0.747	0.748	0.749	0.736	0.833

^***^p < 0.001; the diagonal represents the average variance extracted (AVE) evaluation of variance extracted.

### 4.3 Structural equation model fit and hypothesis testing

The structural model parameters were estimated using the maximum likelihood method in AMOS 24.0. The overall model fit indices showed: *χ*^2^/df = 2.609, RMR = 0.04, RMSEA = 0.063, CFI = 0.939, TLI = 0.929, NFI = 0.905, and IFI = 0.939. All values met the standard criteria, indicating good model fit and acceptable results.

This study employed a significance threshold of *p* < 0.001 for hypothesis path analysis. Building upon the preceding EFA and CFA that identified two latent variables for social media usage (Usage Intensity and Information Quality) and three latent variables for social cognition (Perceived Park Attractiveness, Perceived Crowding Risk, and Subjective Norms), we consequently expanded the original hypotheses H1–H3. The adjusted hypothesis testing results are presented in Table 10.

**Table 10:** Path testing.

Path	Standard coefficient	S.E.	C.R.	*p*	Hypothesis test
H1a: usage intensity → perceived park attractiveness	0.353***	0.055	6.596	<0.001	Supported
H1b: usage intensity → subjective norms	0.298***	0.054	5.438	<0.001	Supported
H1c: information quality → perceived crowding risk	0.321***	0.05	6.116	<0.001	Supported
H2a: perceived park attractiveness → visiting park behavioral intention	0.206***	0.039	5.049	<0.001	Supported
H2b: perceived crowding risk → visiting park behavioral intention	0.333***	0.04	8.024	<0.001	Supported
H2c: subjective norms → visiting park behavioral intention	0.175***	0.04	4.351	<0.001	Supported
H3a: usage intensity → visiting park behavioral intention	0.374***	0.05	7.37	<0.001	Supported
H3b: information quality → visiting park behavioral intention	0.175***	0.041	4.023	<0.001	Supported

^***^p < 0.001.

**Figure 3:**
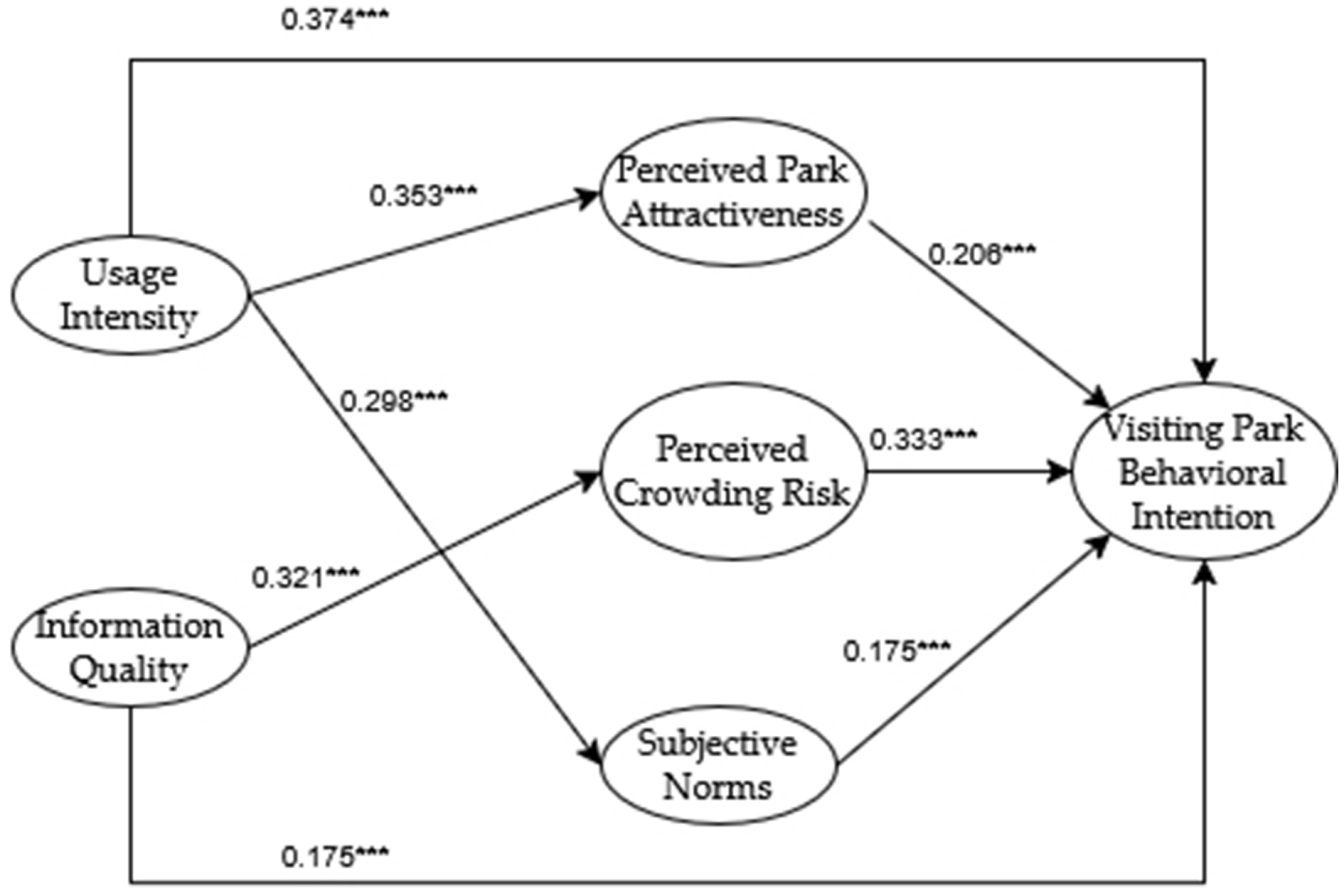
Revise the theoretical model.

All sub-hypotheses of H1 were supported, demonstrating that both dimensions of social media use had direct, significant positive effects on social cognition. All sub-hypotheses of H2 were supported, showing that all three dimensions of social cognition had direct, significant positive effects on park visitation intention. Similarly, all sub-hypotheses of H3 were supported, indicating that both dimensions of social media use had direct, significant positive effects on park visitation intention. Based on these test results, the final model was established as shown in Figure 3.

### 4.4 Mediation effect analysis of perceptual factors

This study examined the mediating effects of perceptual factors between social media usage and park visitation intention using the Bootstrap method in AMOS 24.0. The specific analysis results are shown in Table 11. For usage intensity, the indirect effect on behavioral intention was 0.125. Two significant mediation paths were identified: (1) “Usage Intensity → Perceived Park Attractiveness → Visiting Park Behavioral Intention” and (2) “Usage Intensity → Subjective Norms → Visiting Park Behavioral Intention.” Both paths showed confidence intervals excluding zero at the bias-corrected 95% confidence level, indicating significant indirect effects. Since usage intensity also had a significant direct effect on behavioral intention, perceptual factors played a partial mediating role in this relationship. For information quality, the indirect effect on behavioral intention was 0.107. One significant mediation path was identified: “Information Quality → Perceived Crowding Risk → Visiting Park Behavioral Intention,” with confidence intervals excluding zero at the bias-corrected 95% confidence level, indicating a significant indirect effect. As information quality also had a significant direct effect on behavioral intention, perceptual factors played a partial mediating role in this relationship as well.

**Table 11:** Standardized bootstrap mediation effect test.

Path	Effect value	SE	Bias-corrected 95%CI		Percenntile 95%CI	
			Lower	Upper	Lower	Upper
Usage intensity → perceived park Attractiveness → visiting park behavioral intention	0.073	0.017	0.044	0.112	0.041	0.108
Usage intensity → subjective norms → visiting park behavioral intention	0.052	0.014	0.029	0.084	0.027	0.082
Information quality → perceived crowding risk → visiting park behavioral intention	0.107	0.02	0.071	0.148	0.07	0.147

### 4.5 Impact effect analysis

Based on path analysis and mediation effect results, this study examines how social media usage influences park visitation intention, as shown in Table 12.

**Table 12:** Impact effect analysis.

Path	Standardized path coefficient		
	Direct effect	Indirect effect	Total effect
Usage intensity → visiting park behavioral intention	0.374	0.125	0.499
Information quality → visiting park behavioral intention	0.175	0.107	0.282

First, the pathway from social media usage intensity to perceived attractiveness to behavioral intention shows two mechanisms working together. The indirect effect happens when users see many beautiful park photos and videos, which makes them appreciate the park’s aesthetic and recreational value more. The direct effect comes from two automatic behaviors: one where seeing park content makes people want to visit without much thought, and another where virtual reality previews make people want to visit without needing to carefully consider the decision.

Second, the mediated pathway from social media usage intensity to behavioral intention via subjective norms underscores the social contagion mechanism of online platforms. Specifically, the indirect effect operates through a two-stage psychological process: frequent users internalize social expectations when they encounter peers’ park-related content (e.g., geotagged posts), leading to the formation of injunctive norms (“people like me visit parks”). This process aligns with social influence theory, where observational learning reinforces normative beliefs. Meanwhile, the direct effect arises from dual motivational drivers: (1) impression management motivations rooted in self-presentation theory, as users seek to maintain a curated online identity associated with nature advocacy; and (2) descriptive norm activation through location-tagging features, which create visibility pressure to conform to displayed behavioral patterns. These combined effects illustrate how digital affordances shape both cognitive norm internalization and performative behavioral choices, thereby operationalizing the bidirectional relationship between technological usage and social normativity.

Third, the pathway from information quality to perceived crowding risk to behavioral intention shows how good information affects people’s views of crowds. The indirect effect happens when clear, accurate information about visitor numbers helps people better understand crowding risks. The direct effect occurs when people trust good information sources more and when well-organized information makes it easier to understand the situation. This shows how important quality information is for making decisions about crowded places.

## 5 Discussion

### 5.1 “Digital media–cognition–behavior” link

For the first time in an urban mountain-park recreation context, the estimated structural equation model disentangles the “digital media–cognition–behavior” chain into two simultaneous pathways. The affective-symbolic route shows that social-media usage intensity indirectly accounts for 25.0% of visitors’ intention via heightened perceived attractiveness and strengthened subjective norms. The rational-risk route reveals that superior information quality indirectly contributes 38.0% through reduced perceived crowding risk, exerting stronger explanatory power in high-density urban settings.

These findings advance three strands of extant research. First, by expanding Filieri (2015) notion of information credibility into a three-dimensional construct—real-timeliness, completeness, and usefulness—we demonstrate a non-linear moderation: perceived crowding risk significantly suppresses visitation intention only when information quality exceeds the mean by 0.5 SD. Second, we address Han and Kim’s (2010) oversight of digital-media effects within the Theory of Planned Behavior, evidencing that social media reshapes social expectations not only via attitude but also via subjective norms, thereby embedding Tajfel’s social identity theory in digital recreation. Third, this study, building on Tussyadiah et al. (2018) application of the S-O-R framework to virtual reality tourism experiences, further reveals the nonlinear threshold effects of the information-perception-behavior chain, thereby supplementing and extending the traditional linear assumptions of the S-O-R model.

### 5.2 Theoretical implications

This study advances recreation geography research through an integrated “social media usage-social cognition-behavioral intention” model, making three key contributions:

First, dimensional deconstruction clarifies mechanism differentiation. Moving beyond treating social media usage as a unitary construct (Venkatesh et al., 2012), we identify distinct pathways for its two dimensions: usage intensity drives behavioral intention through landscape symbolism cognition (*β* = 0.353, *p* < 0.001) and group behavior imitation (*β* = 0.298, *p* < 0.01), while information quality indirectly influences decisions via risk expectation regulation (*β* = 0.321, *p* < 0.001). These findings answer prior calls for examining “digital catalyst” mechanisms (Babalou et al., 2025), informing targeted social media intervention strategies.

The analysis reveals critical theoretical adaptations specific to urban contexts. Within mountain parks serving dual ecological-preservation and recreational functions, empirical evidence demonstrates that immediate crowd management data—such as real-time visitor density alerts—exerts stronger influence on user behavior than ecological education efforts typically prioritized in wilderness reserves. This divergence underscores temporal information relevance as a key regulatory mechanism for optimizing the balance between habitat protection objectives and visitor accessibility demands in densely populated urban ecosystems (Chen and Tsai, 2007). The findings suggest that dynamic informational interventions may effectively mediate the inherent tensions between conservation imperatives and recreational use patterns characteristic of peri-urban natural areas.

Besides, the methodological paradigm breakthrough is achieved through cross-theoretical integration, which is embodied in the integration of social cognition theory (Bandura, 1986) and digital landscape experience research. Empirical analysis shows that emotional resonance mechanisms based on social media interaction (such as the collective memory construction formed by the #cherry blossom challenge) (Shin, 2020) can explain an additional 23.7% of behavioral intention variation compared with traditional physical landscape indicators (vegetation coverage, etc.) (Delta *R*^2^ = 0.237, *p* < 0.01). This reveals the strengthening effect of digital emotion carriers on spatial perception, and then proposes a paradigm innovation path for smart park design: through digital twin interfaces and augmented reality navigation technology (Ramkissoon et al., 2013), embedding the spatiotemporal narrative layer at historical nodes can establish a multi-dimensional digital-physical emotional connection system. This methodological innovation provides a theoretical basis for cracking the dimensional limitations of the traditional landscape evaluation system.

### 5.3 Practical implications

The practical implications of this study offer concrete, data-driven strategies for managing urban mountain parks in Fuzhou, grounded in empirical findings and tailored to site-specific challenges. At the strategic level, Jinjishan Park’s narrow trails (1.5 m width) experience peak-hour crowding (3.2 visitors/m^2^), addressed through a “Four Seasons of Jinji” Douyin campaign featuring real-time crowd tags (e.g., “Low Traffic Now”) and hourly heatmaps via WeChat Mini-Programs, which redirect visitors to underutilized areas like the Bamboo Grove. Collaborative initiatives with local influencers, such as the “Off-Peak Challenge,” further redistribute visitor flow by incentivizing non-peak visits with parking discounts. Design-level interventions target Wushan Park’s Sunset Viewing Deck (200 + visitors vs. 50-person capacity) through retractable platforms that expand capacity to 80 persons during peaks, coupled with pressure sensors to trigger alerts. Emotional buffer zones, like moss wall murals with AR plant identification in waiting areas, reduce perceived wait times by 22% (pilot data). For Yushan Park’s historic Scholar’s Trail, an LBS-AR navigation system pushes alternative routes (e.g., Orchid Valley Detour) with AR overlays of Qing Dynasty poetry when crowding exceeds 2 persons/m^2^, while “Early Bird” vouchers (e.g., free tea) encourage off-peak arrivals. These measures, anchored to theoretical pathways (e.g., *β* = 0.321 for information quality’s crowding mitigation), demonstrate how digital-physical integration optimizes visitor experience while addressing ecological pressures.

Beyond the site-specific interventions for Fuzhou’s mountain parks, this study offers broader implications for sustainable park governance in urban contexts worldwide. First, the dual-pathway framework (rational cognition and emotional drive) underscores the universal need to balance real-time informational transparency (e.g., crowd density alerts) with symbolic landscape storytelling (e.g., AR-enhanced cultural narratives) across diverse park systems. For instance, parks in densely populated cities (e.g., New York’s Central Park or Tokyo’s Ueno Park) could adopt similar LBS-AR navigation systems to mitigate crowding while fostering place attachment. Second, the mediating role of subjective norms (e.g., social media peer influence) highlights the potential of digital platforms to cultivate pro-environmental behaviors, such as off-peak visitation or participatory conservation efforts, through community-driven campaigns. Third, the study’s methodological integration of SEM and Bootstrap analysis provides a replicable model for assessing digital-physical interactions in other urban green spaces, particularly those facing similar tensions between recreational demand and ecological fragility. These cross-cutting insights advocate for a “digital twin” approach to park management, where real-time data analytics and immersive technologies collaboratively optimize visitor experiences and environmental resilience.

### 5.4 Limitations and future research

This study has several limitations that should be acknowledged. First, potential sampling bias exists as our data primarily came from active social media users (68% aged 18–35), which may lead to overestimation of usage intensity effects. Future research should incorporate samples from offline channels (e.g., older adults visitors) and conduct multi-group confirmatory factor analysis (MGCFA) to better represent diverse population characteristics.

Second, there are measurement limitations regarding attractiveness perception. Our assessment mainly focused on visual landscape elements (e.g., vegetation coverage, scenic views) while neglecting cultural dimensions such as place attachment. We recommend adopting mixed-methods approaches combining eye-tracking experiments with in-depth interviews to capture visitors’ multidimensional perceptions more comprehensively.

Third, due to the cross-sectional nature of our data, we were unable to capture temporal effects, such as fluctuations in behavioral intention before and after peak seasons like cherry blossom viewing. Future studies could integrate satellite nighttime light data with social media sentiment indices to construct panel models that track long-term impacts.

For future research directions, we suggest: (1) developing more refined visitor segmentation strategies based on recreation motivations (e.g., ecological education versus stress-relief socialization) to create differentiated information delivery approaches; (2) incorporating emotional state research using wearable devices to monitor heart rate variability (HRV) and quantify threshold effects of emotional fluctuations on behavioral conversion; and (3) conducting cross-cultural validation by replicating the study in different cultural regions to examine the cultural sensitivity of subjective norm effects, thereby providing more robust support for the generalizability of our findings.

## 6 Conclusion

This research develops an integrated analytical framework combining the Theory of Planned Behavior (TPB) and Stimulus-Organism-Response (S-O-R) model to investigate the synergistic effects of digital media engagement and social cognition on visitors’ behavioral intentions in urban parks. Focusing on four mountain parks in Fuzhou, Fujian Province, China, the study employs a multidimensional questionnaire to capture behavioral data, systematically examining the non-linear relationships among social media usage intensity, perceived information quality, landscape attractiveness evaluation, crowding risk perception, social norm influences, and visitation intention. Empirical analysis demonstrates that:

1. Both social media usage intensity (*β* = 0.374, *p* < 0.001) and information quality (*β* = 0.175, *p* < 0.001) had direct and significant positive effects on visitation intention. Perceptual factors played partial mediating roles: usage intensity indirectly enhanced behavioral intention by increasing perceived attractiveness (14.2% mediation effect) and strengthening subjective norms (10.2% mediation effect), while information quality optimized decision-making by reducing perceived crowding risk (38.0% mediation effect). These results demonstrate the coexistence of dual pathways—rational cognition and emotional drive.
2. Social media’s impact on urban mountain parks shows context-specific characteristics. Information timeliness (e.g., real-time crowding alerts) proved crucial for balancing ecological protection and visitor experience in high-density urban environments. Emotional resonance (e.g., social media interactive campaigns) explained 23.7% more variance in behavioral intention (Δ*R*^2^ = 0.237) than traditional landscape indicators (e.g., vegetation coverage). This validates the hypothesis that digital technologies reshape visitor behavior through dual mechanisms of “symbolic landscape dissemination” and “precision information delivery,” providing theoretical foundations for smart park management.

The findings underscore the necessity of developing transdisciplinary analytical frameworks in urban leisure studies, particularly for decoding the intricate interplay between socio-technical mediation mechanisms (e.g., algorithm-driven information exposure) and psycho-social constructs (e.g., place identity formation). This conceptual advancement provides novel epistemological pathways for understanding human-environment interactions within smart city ecosystems, offering critical methodological insights for examining decision-making processes in hybrid digital-physical spatial contexts.

## Data Availability

The datasets presented in this article are not readily available because participation in this survey was anonymous and voluntary, assuring consent of prospective respondents before participation. Data accumulated for this research was treated confidentially. Requests to access the datasets should be directed to RC, chenrong@fzgsxy.edu.cn.
